# Improved empirical wavelet transform (EWT) and its application in non-stationary vibration signal of transformer

**DOI:** 10.1038/s41598-022-22519-z

**Published:** 2022-10-20

**Authors:** Ruizheng Ni, Ruichang Qiu, Zheming Jin, Jie Chen, Zhigang Liu

**Affiliations:** School of Electrical Engineering, Beijing Jiaotong University, Beijing, 100044 China

**Keywords:** Engineering, Energy infrastructure, Energy grids and networks, Power distribution

## Abstract

The resonant frequency of the transformer contains information related to its structure. It is easier to identify the resonance frequency in the vibration signal during the hammer test and power on than in the operation of the transformer, because the vibration caused by the load current does not need to be considered during the hammer test and power on. Therefore, an analysis method with simple calculation, fast calculation speed and easy real-time monitoring is needed to deal with these two non-stationary vibrations. Vibration monitoring can understand the health status of transformer in real time, improve the reliability of power supply and give early warning in the early stage of faults. A new frequency domain segmentation method is proposed in this paper. This method can effectively process the vibration signal of transformer and identify its resonant frequency. Eleven different load states are set on the transformer. The method proposed in this paper can extract the resonant frequency of the transformer from the hammering test signal. Compared with the original empirical wavelet transform method, this method can divide the frequency domain more effectively, has higher time–frequency resolution, and the running time of the modified method is shortened from 80 to 2 s. The universality of this method is proved by experiments on three different types of transformers.

## Introduction

Due to the improvement of power supply stability requirements, there are more and more researches on transformer health assessment. Common fault diagnosis methods of transformer include regular inspection, dissolved gas analysis^1^, vibration monitoring^2,3^, partial discharge monitoring^4^, ultrasonic measurement^5^, frequency–response analysis^6^ and other methods. Compared with the other methods, vibration measurement has the advantages of convenient installation, less environmental interference and low cost. It is applicable to almost all types of transformers.

The vibration of the transformer mainly comes from the magnetostriction and magnetic forces. Through the real-time monitoring of transformer vibration, the relationship between abnormal vibration and internal faults of transformer will be established, which is helpful to arrange preventive maintenance in time and improve the service life of transformer. For example, when the tightening bolt of transformer core is loose, that is, the air gap between iron core changes, it will significantly increase the vibration of transformer^7^, in addition, loose bolts also reduce the ability of the transformer to resist external shocks. The mechanical performance degradation of the transformer was tracked through multi-channel vibration measurement in^8^. In^9^, the vibration data on the transformer on-load tap changer is obtained to realize the identification of early equipment faults, and the self-organizing mapping (SOM) is used to evaluate the status of the on-load tap changer online. In^10^, the method of monitoring winding deformation by transformer box vibration was studied, this method takes into account the vibration generated by different parts of transformer, and analyzes the influence of temperature on vibration generation, superposition and transmission.

The vibration frequency of transformer depends on the resonance frequency and external excitation. The external excitation mainly includes the voltage, current and working environment, these factors can be measured during the operation of the transformer. Resonance frequency is the internal factor that determines the vibration frequency of the transformer. It is determined by the transformer structure and does not change with the change of external excitation. It can be obtained by hammering test. The closer the vibration component is to the resonance frequency, the more likely it will cause the transformer resonance. Resonance is very harmful, which will lead to violent vibration of the transformer, resulting in the loosening of bolts and the falling off of cushion blocks. In addition, the structure can be tracked by monitoring the resonance frequency of the transformer, and the fault diagnosis of the transformer can be realized by analyzing the change of the transformer structure. In paper^11^, the resonance frequency of transformer was calculated by pseudo spectral method. The relationship between transformer vibration frequency with the voltage and current harmonics was deduced in paper^12^. The influence of vibration on the operation of the large transformer and the vibration reduction measures to avoid resonance under electromagnetic force excitation were studied in^13^, and a prototype power transformer with very low noise was developed, with full load capacity of 200MVA and noise level less than 65 dB. The nonlinear model of transformer was built by Fourier neural network composed of nonlinear elements and a linear dynamic block, and the effect of vibration prediction and the system parameter extraction were verified by testing on several power transformers^14^.

There are many analytical methods to deal with vibration data. Fourier analysis is a simple and effective analysis method, but Fourier transform method cannot display time–frequency information at the same time^15^. The paper^16^ proposed a simplified permutation entropy algorithm which is used to calculate the vibration features of the converter transformer. Compared with the traditional permutation entropy algorithm, the algorithm has the advantages of stable classification, high flexibility and fast computing speed. The wavelet transform method^17,18^ also has many applications in transformer vibration monitoring. Complex Morlet wavelet was used to process the free vibration data of transformer in^17^, the improved Crazy Climber algorithm was used to extract the wavelet ridges of the time–frequency spectrogram, and the first four order resonance frequencies and damping ratios of the transformer winding were obtained. A new method for mechanical fault diagnosis of transformer cores and windings based on frequency band—energy distribution was proposed in^18^. The transformer mechanical faults were diagnosed online by the energy distribution in each frequency band of the real-time vibration data. The improved empirical mode decomposition algorithm was applied to the fault index extraction of vibration data on the transformer on-load tap change^19^. EWT was first introduced by Professor Jérôme Gilles^20^, it is equivalent to a series of band-pass filter combinations, and the original signal is decomposed into several signal combinations in different frequency domains. Papers^21,22^ introduced the application of EWT in seismic data, and^22^ proposed an improved EWT method based on scale-space representation. Paper^23^ introduced the application of EWT in two-dimensional image recognition. An improved adaptive Morlet wavelet transform and its application in vibration data of gearbox were introduced in paper^24^. EWT also has some applications in transformers. In paper^25^, a fault diagnosis method based on EWT and salp swarm algorithm was proposed to diagnose different fault states of transformers. In paper^26^, the EWT method was combined with multi-scale entropy, and the counting times were reduced by selecting the wavelet components highly correlated with the original signal.

EWT is selected from many non-stationary signal processing methods mainly because it can improve the resolution of target frequency component by flexibly setting frequency domain segmentation boundary, and EWT frequency domain segmentation is based on Fourier transform, the two methods are partially overlap, so the signal processing process is a progressive relationship, which can reduce the burden of calculation; more importantly, by setting the boundary near the abnormal target frequency component according to the Fourier result, the target component can be analyzed in depth to determine the change time of the target frequency component, it is very important for transformer fault diagnosis. The spectrum division method proposed in this paper combines factors such as frequency domain extremum and envelope, which can not only track and analyze the target components, but also reasonably divide the spectrum range. Different from paper^26^, this paper does not combine other methods, but directly improves the spectrum division principle on EWT, simplifies the calculation process, and improves the adaptability. Compared with the traditional method based on the scale plane, it eliminates the step of creating the scale plane, greatly improves the calculation speed, and is more suitable for real-time analysis and monitoring.

## Transformer vibration analysis

According to the location of vibration, the vibration of transformer can be divided into iron core vibration, winding vibration and cooling equipment vibration. According to the determinants of vibration frequency, it can be divided into vibration determined by resonance frequency and vibration determined by excitation frequency.

## Vibration of winding and iron core

The vibration of transformer winding is caused by the interaction between current and magnetic flux leakage in the winding, the vibration force of coil *F*_*w*_ is proportional to the square of current *I*, as shown in Eq. (1). The vibration of transformer core is caused by magnetostriction and magnetic force, the vibration force of iron core *F*_*c*_ is proportional to the square of voltage *U*, as shown in Eq. (2).

1 $$ F_{w} \propto I^{2} $$

2 $$ F_{c} \propto U^{2} $$

The current and voltage in the transformer are sine waves mixed with a small amount of harmonics, which can be expressed as Eqs. (3) and (4), *I*_0_ and *U*_0_ represent the DC components of current and voltage respectively and *I*_*n*_ and *U*_*n*_ represent the fundamental components and harmonic components of current and voltage respectively.

3 $$ I = I_{0} + \sum\limits_{n = 1}^{\infty } {I_{n} \cos \left( {n\omega t + \varphi_{n} } \right)} $$

4 $$ V = V_{0} + \sum\limits_{n = 1}^{\infty } {V_{n} \cos \left( {n\omega t + \varphi_{n} } \right)} $$

Combine Eq. (3) into Eq. (1) to obtain

5 $$ \begin{gathered} F_{w} \propto \left( {I_{0} + \sum\limits_{n = 1}^{\infty } {I_{n} \cos \left( {n\omega t + \varphi_{n} } \right)} } \right)^{2} \hfill \\ \, \propto I_{0}^{2} + 2I_{0} \sum\limits_{n = 1}^{\infty } {I_{n} \cos \left( {n\omega t + \varphi_{n} } \right)} \hfill \\ \, + \left( {\sum\limits_{n = 1}^{\infty } {I_{n} \cos \left( {n\omega t + \varphi_{n} } \right)} } \right)^{2} \hfill \\ \end{gathered} $$

The third part of Eq. (5) can be expressed as Eq. (6), *p* and *q* is a number between 1 and combinatorial numbers C_*n*_^2^.

6 $$ \begin{aligned} \left( {\sum\limits_{n = 1}^{\infty } {I_{n} \cos \left( {n\omega t + \varphi_{n} } \right)} } \right)^{2} & = \sum\limits_{n = 1}^{\infty } {\left( {I_{n} \cos \left( {n\omega t + \varphi_{n} } \right)} \right)^{2} } \\ & \quad + \sum\limits_{p,q \in [1 \cdots n]}^{{C_{n}^{2} }} {2\left( {I_{p} \cos \left( {p\omega t + \varphi_{p} } \right)} \right)} \left( {I_{q} \cos \left( {q\omega t + \varphi_{q} } \right)} \right) \\ \end{aligned} $$

According to the transformation rules of trigonometric equation, the second part of Eq. (6) can be expressed as

7 $$ \begin{aligned} 2\left( {I_{p} \cos \left( {p\omega t + \varphi_{p} } \right)} \right)\left( {I_{q} \cos \left( {q\omega t + \varphi_{q} } \right)} \right) & = I_{p} I_{q} \left( {\cos \left( {p\omega t + \varphi_{p} + q\omega t + \varphi_{q} } \right)} \right) \\ & \quad + I_{p} I_{q} \left( {\cos \left( {p\omega t + \varphi_{p} - q\omega t - \varphi_{q} } \right)} \right) \\ \end{aligned} $$

The transformer vibration frequency will include the DC component, the harmonic frequency component, the double frequency of each harmonic component, the sum of any two harmonic components and the difference of any two harmonic components, as shown in Table 1. When the transformer works, the current and voltage are close to the ideal sine waves with low harmonic contents, and it can be seen from Table 1 that the amplitude of each excitation force is in the same order of magnitude, so the vibration amplitude caused by harmonics mainly depends on the position of the resonance frequency point, this is also helpful to determine the distribution of resonance frequency by observing the distribution of vibration frequency.

**Table 1:** Frequency component and amplitude of excitation force.

Amplitude	Frequency
*I*_0_^2^	0
2*I*_0_*I*_*n*_	*nω*
*I*_*n*_^2^/2	2*nω*
*I*_*p*_* I*_*q*_	*(p* + *q*) *ω*
*I*_*p*_* I*_*q*_	*(p—q*) *ω*

## Influence of resonance frequency

During the operation of transformer, the vibration amplitude and vibration speed are very small, which belongs to a micro amplitude system. Therefore, the transformer vibration system can be regarded as a linear system by taking only the first-order term of Taylor series and omitting the higher-order term. The motion equation of forced vibration of single-freedom linear system is shown in Eq. (8), *m*, *c*, *k* are mass, damping coefficient and elastic coefficient respectively.

8 $$ m\frac{{\partial^{2} x}}{\partial t} + c\frac{\partial x}{{\partial t}} + kx = F $$

Equation (8) can be rewritten as

9 $$ \frac{{\partial^{2} x}}{\partial t} + 2\xi \omega_{n} \frac{\partial x}{{\partial t}} + \omega_{n}^{2} x = \frac{F}{m} $$

The parameters in Eq. (9) are defined as follows:

10 $$ \omega_{n} = \sqrt{\frac{k}{m}}  $$

11 $$ \xi = \frac{c}{{2m\omega {}_{n}}} $$

In Eqs. (10) and (11), *ω*_*n*_ is the resonance frequency and *ξ* is the damping rate. Because the transformer system is approximately a linear system, the vibration under each excitation satisfies the superposition theorem. For the convenience of analysis, it is assumed that the excitation force *F* = *f*_*n*_cos*ωt*. Substituting into Eq. (9) to obtain

12 $$ \begin{aligned} x(t) & = Xe^{{ - \xi \omega_{n} t}} \cos \left( {\omega_{d} t - \phi } \right) \\ & \quad + \frac{{f_{n} }}{{m\sqrt {\left( { - 2\xi \omega \omega_{n} } \right)^{2} + \left( {\omega_{n}^{2} - \omega^{2} } \right)^{2} } }}\cos \left( {\omega t - \varphi } \right) \\ \end{aligned} $$

The parameters in Eq. (12) are defined as follows:

13 $$ \begin{aligned}    & \omega _{d}  = \sqrt {1 - \xi ^{2} } \omega _{n}  \\     & X = \sqrt {x_{0}^{2}  + \left( {\frac{{v_{0}  + \xi \omega _{n} x_{0} }}{{\omega _{d} }}} \right)^{2} }  \\  \end{aligned}  $$

14 $$ \begin{gathered} \phi = \arctan \frac{{v_{0} + \xi \omega_{n} x_{0} }}{{\omega_{d} x_{0} }} \hfill \\ \varphi = \arctan \frac{{2\xi \omega \omega_{n} }}{{\omega_{n}^{2} - \omega^{2} }} \hfill \\ \end{gathered} $$

**Figure 1:**
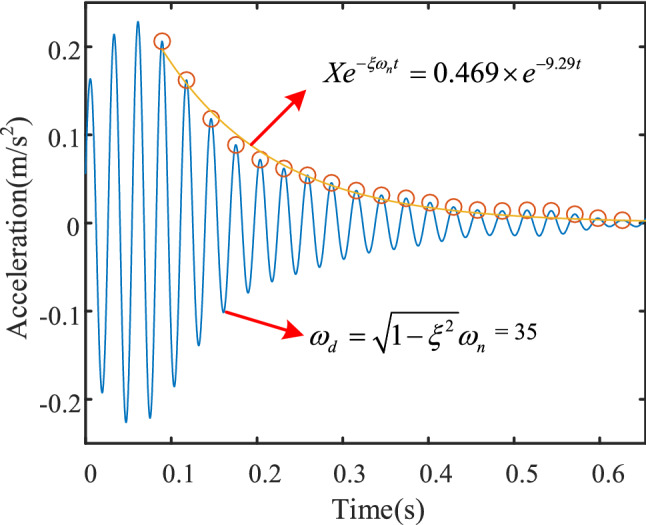
Fitting of resonance waveform.

It can be seen from Eq. (12) that the first item in the equation will gradually attenuate and set to zero under the action of transformer damping. The waveform of specific frequency in hammering test can be extracted by improved EWT, and the relevant parameters (*X*, *ξ*, *ω*_*n*_) can be obtained by fitting the waveform envelope. Figure 1 shows the attenuation process of 37 Hz frequency component in hammering test, which corresponds to the resonance frequency component of 37 Hz in Fig. 6. The values of relevant parameters are obtained by parameter fitting, as shown in (15)

15 $$ \begin{gathered} X = 0.469 \hfill \\ \xi = 0.251 \hfill \\ \omega_{n} = 37 \hfill \\ \end{gathered} $$

For the second part of Eq. (12), the vibration of transformer under different frequency excitation force is still the response of corresponding frequency, but the vibration amplitude is affected by transformer structure coefficient and resonance frequency. Let *p* be as shown in Eq. (16), *p* is a nonnegative variable. The smaller *p* is, the greater the vibration amplitude is.

16 $$ p = \left( {2\xi \omega \omega_{n} } \right)^{2} + \left( {\omega_{n}^{2} - \omega^{2} } \right)^{2} $$

Equation (16) can be transformed into

17 $$ p = \omega^{4} + \left( {4\xi^{2} - 2} \right)\omega_{n}^{2} \omega^{2} + \omega_{n}^{4} $$

*p* is a univariate quartic equation with *ω* as the variable, then

18 $$ p^{^{\prime}} = 4\omega^{3} + 2\left( {4\xi^{2} - 2} \right)\omega_{n}^{2} \omega $$

*p*′ is a univariate cubic equation, let *p*′ = 0, we will get

19 $$ \begin{aligned}    & \omega _{1}  = 0 \\     & \omega _{2}  = \sqrt {\left| {1 - 2\xi ^{2} } \right|} \omega _{n}  \\     & \omega _{3}  =  - \sqrt {\left| {1 - 2\xi ^{2} } \right|} \omega _{n}  \\  \end{aligned}  $$

Therefore, *p* has the minimum value at *ω*_2_, that is, in Eq. (12), the closer *ω* is to *ω*_2_, the greater the amplitude of *x*(*t*), *x*(*t*) has the maximum vibration amplitude when *ω* = *ω*_2_.

## Transformer vibration data processing method

### Empirical wavelet transform

20 $$ \phi_{N} \left( \omega \right) = \left\{ {\begin{array}{*{20}l} 1 \hfill & {{\text{if}}\;\left| \omega \right| \le \omega_{N} - \tau_{N} } \hfill \\ {\cos \left[ {\frac{\pi }{2}\beta \left( {\frac{1}{{2\tau_{n} }}\left( {\left| \omega \right| - \omega_{N} + \tau_{N} } \right)} \right)} \right]} \hfill & {{\text{if}}\;\omega_{N} - \tau_{N} \le \left| \omega \right| \le \omega_{N} + \tau_{N} } \hfill \\ 0 \hfill & {{\text{otherwise}}} \hfill \\ \end{array} } \right. $$

EWT is essentially a wavelet transform that can flexibly set the segmentation boundary^20^. The core of EWT method lies in the division of frequency domain. The spectrum results vary greatly in different applications, but the purpose of spectrum segmentation is the same, which is to highlight the change process of target frequency component. The empirical scaling function and the empirical wavelets are shown by the Eqs. (20) and (21), respectively.

21 $$ \varphi_{N} \left( \omega \right) = \left\{ {\begin{array}{*{20}l} 1 \hfill & {{\text{if}}\;\omega_{N} + \tau_{N} \le \left| \omega \right| \le \omega_{N + 1} - \tau_{N + 1} } \hfill \\ {\cos \left[ {\frac{\pi }{2}\beta \left( {\frac{1}{{2\tau_{N + 1} }}\left( {\left| \omega \right| - \omega_{N + 1} + \tau_{N + 1} } \right)} \right)} \right]} \hfill & {{\text{if}}\;\omega_{N + 1} - \tau_{N + 1} \le \left| \omega \right| \le \omega_{N + 1} + \tau_{N + 1} } \hfill \\ 0 \hfill & {{\text{otherwise}}} \hfill \\ \end{array} } \right. $$

The function *β*(*x*) shall meet the following conditions:

22 $$ \beta \left( x \right) = \left\{ {\begin{array}{*{20}l} 1 \hfill & {{\text{if}}\;x \ge 1} \hfill \\ {1 - \beta \left( {1 - x} \right)} \hfill & {{\text{if}}\;0 < x < 1} \hfill \\ 0 \hfill & {{\text{if}}\;x \le 0} \hfill \\ \end{array} } \right. $$

The *τ*_*N*_ = *γω*_*N*_, and if *γ* < min[(*ω*_*N*+1_ − *ω*_*N*_)/(*ω*_*N*+1_ + *ω*_*N*_)], we will get a tight frame. Then the signal can be reconstruction by:

23 $$ f\left( t \right) = W_{f}^{\varepsilon } \left( {0,t} \right) * \phi_{1} \left( t \right) + \sum {W_{f}^{\varepsilon } \left( {N,t} \right) * \varphi_{N} \left( t \right)} $$

### Improved frequency domain segmentation method

From the previous analysis, it can be found that the focus of EWT is to determine the frequency domain segmentation boundary. The processing of different types of signals needs to combine their unique characteristics. The components of transformer vibration data have the following characteristics.

1. During transformer operation, the main component in the frequency domain is usually an integral multiple of 50 Hz (the electrical frequency is 50 Hz). Under normal circumstances, the 100 Hz component should have the largest amplitude, but due to the influence of resonance frequency, the 50 Hz frequency doubling component closest to the resonance frequency point may be the largest amplitude component.
2. When the transformer is not working, the amplitude of environmental interference is below 0.003 m/s^2^, and the corresponding value in the Fourier transform result is 0.003*16,384/2 ≈ 25, the sampling frequency of vibration signal is 16384 Hz. In the analysis of the Fourier result of vibration signal, the signal below 25 can be ignored.
3. Continuous high amplitude components may occur during hammering test or transformer state switching. These components will increase the difficulty of frequency domain segmentation.
4. Some frequency components with low amplitude will appear near the high amplitude components. We should ignore the influence of these low amplitude components when dividing the frequency domain.
5. In the process of transformer fault diagnosis, the high amplitude components, the emerging frequency components and the components with great change are important, the latter two parts are collectively referred to as abnormal frequency components. It is necessary to set boundaries near these frequency components.

**Figure 2:**
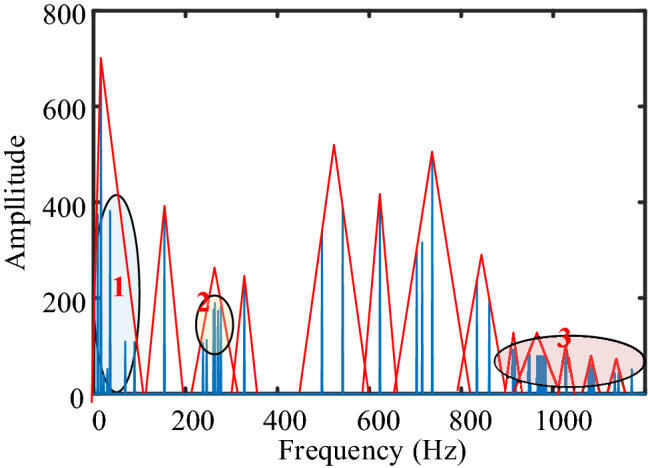
Spectrum of transformer hammering test.

Figure 2 shows the spectrum of transformer hammering vibration, the blue vertical line in the figure represents the maximum component in the spectrum, the red triangles represent the ideal division results of the spectrum, and each red triangle represents a division area. The spectrum situation during state switching is similar to that of hammering test. When dividing this kind of spectrum, we should pay attention to the following points.

1. The low amplitude component near the high amplitude corresponds to area 1 in Fig. 2.
2. Adjacent components with similar amplitude correspond to area 2 in Fig. 2.
3. The low amplitude component corresponds to area 3 in Fig. 2.
4. The division boundary cannot fall on the maximum component.
5. The boundary of the high amplitude region should be more dense, such as the 400-800 Hz range in Fig. 2, and the boundary of the low amplitude region should be more sparse, such as the 900–1100 Hz range.

**Figure 3:**
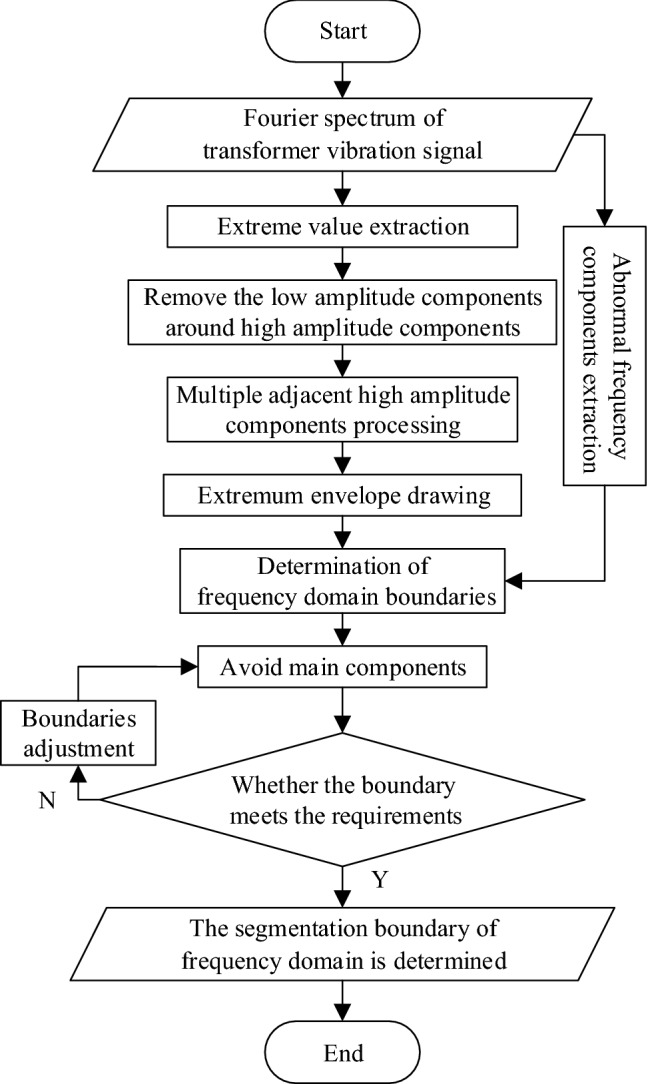
Improved frequency domain segmentation process.

The proposed frequency domain segmentation method is shown in Fig. 3. The procedure of the proposed frequency domain segmentation method includes the following four steps.

- Step 1: The Fourier spectrum of the analyzed signal is analyzed to obtain the abnormal frequency components of the signal. And the frequency domain extremums are extracted.
- Step 2: Removal of interference signals that affecting boundary segmentation, including the clutter low amplitude components around the high amplitude component, the multiple adjacent components with similar high amplitudes, and the components with small amplitude in frequency domain.
- Step 3: The dividing boundary is determined according to the envelope of the remaining main frequency components after removing the interference signals, and the abnormal components in Step 1 needs to be analyzed emphatically.
- Step 4: Check the segmentation boundary to prevent it from falling on the main components of the frequency domain, otherwise these components will be attenuated in the subsequent analysis process.

We use Eq. (24) to remove the low amplitude components around high amplitude components, and use formula (25) to deal with the multiple adjacent high amplitude components. *f*(*a*) and *f*(*b*) represent the amplitudes of the two components in the frequency domain, and *a* and *b* represent the frequencies of the two components. Equations (24) and (25) are two simple and effective methods to remove interference components. The selection of relevant coefficients in the formula is very important. The transformer used in this paper is a dry-type transformer with a capacity of 100 kVA. During the experiment, *k*_*p*_ and *r* are taken as 20, 100 respectively, *k*_*q*_ and *s* are taken as 0.15 and 30 respectively. The above four parameters are empirical parameters in the experimental process. Because the working frequency of the transformer is 50 Hz, for a maximum frequency component, the left and right sides are 50 Hz, and the frequency bandwidth is 100 Hz, that is, *r* = 100. When considering the adjacent frequency components, the bandwidth take half of 50, 50/2 = 25, that is, *s* = 25, *r* and *s* are two parameters only related to the power supply frequency of the transformer, the determination of *k*_*p*_ and *k*_*q*_ should be combined with the vibration of the transformer. In the experiment, the maximum amplitude of frequency domain is basically about 500 (as shown in Figs. 5 and 7), 500/25 = 20, that is, *k*_*p*_ = 20, (25 here refers to the value of ambient noise in Step3, 0.003*16,384/2 ≈ 25), and *k*_*q*_ = 0.15 is obtained from multiple experiment.

24 $$ \left\{ {\begin{array}{*{20}l}    {f(a) - f(b) > k_{p} \left| {a - b} \right|} \hfill  \\    {\left| {a - b} \right| < r} \hfill  \\   \end{array} } \right. $$

25 $$ \left\{ {\begin{array}{*{20}l}    {f(a) - f(b) < k_{q} \left( {f(a) + f(b)} \right)} \hfill  \\    {\left| {a - b} \right| < s} \hfill  \\   \end{array} } \right. $$

For the frequency component with sudden change, we can add segmentation lines on both sides. In order to prevent the frequency segmentation from being too dense or too sparse, the number and position of boundary lines can be flexibly adjusted according to the mean value of frequency components within this range. If the division is too dense, the average value of the two boundaries or the boundary with low frequency amplitude or the boundary near the target frequency can be taken. The last method can improve the resolution of the target frequency amplitude. For a region with sparse division, a division boundary line may be appropriately added at some frequency domain minimum values.

## Analysis of non-stationary vibration data

**Figure 4:**
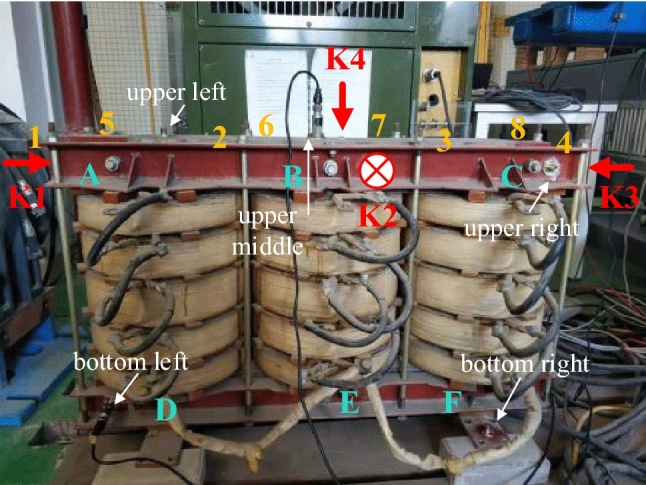
Vibration test transformer.

The vibration sensor adopts CA-YD-188T piezoelectric acceleration sensor of Jiangsu Lianneng company, with sensitivity of 500 mv/g, frequency range of 0–5000 Hz, measurement range of ± 10 g, impact limit of 2000*g* and working temperature range of − 40 to 120 ℃. The vibration acquisition instrument uses the high dynamic range acquisition unit of Dewesoft company. As shown in Fig. 4, the experimental transformer has 14 fastening bolts, 6 transverse bolts (A–F) and 8 longitudinal bolts (1–8). There are five installation positions (upper left, upper middle, upper right, bottom left, bottom right) of vibration sensors on the transformer. The hammering test consists of four times in each group, standing on the side of the high-voltage winding and facing the transformer, the first hammering (K1) is from left to right at the top of the transformer, the second hammering (K2) is from front to back in the direction of sight, the third hammering (K3) is from right to left at the top of the transformer, and the fourth hammering (K4) is downward from the middle of the top. The method proposed in this paper is mainly used to analyze the transient vibration of the transformer, including the vibration of the transformer under power on, power off and load switching between different loads, as shown in Table 2. The methods proposed in this paper are applicable to the transient situation when the state is switched, but only shows the results of hammer test, power on and load switching from 60 to 100 kW.

**Table 2:** The situation of load switching.

State	Load
Power on	0 kW + 0 kvar
Increase active load	30 kW + 0 kvar
	60 kW + 0 kvar
	100 kW + 0 kvar
Increase reactive load	100 kW + 20kvar
	100 kW + 70kvar
Reduce reactive load	100 kW + 20kvar
	100 kW + 0 kvar
Reduce active load	60 kW + 0 kvar
	30 kW + 0 kvar
	0 kW + 0 kvar
Power off	0 kW + 0 kvar

### Vibration analysis of transformer hammering test

**Figure 5:**
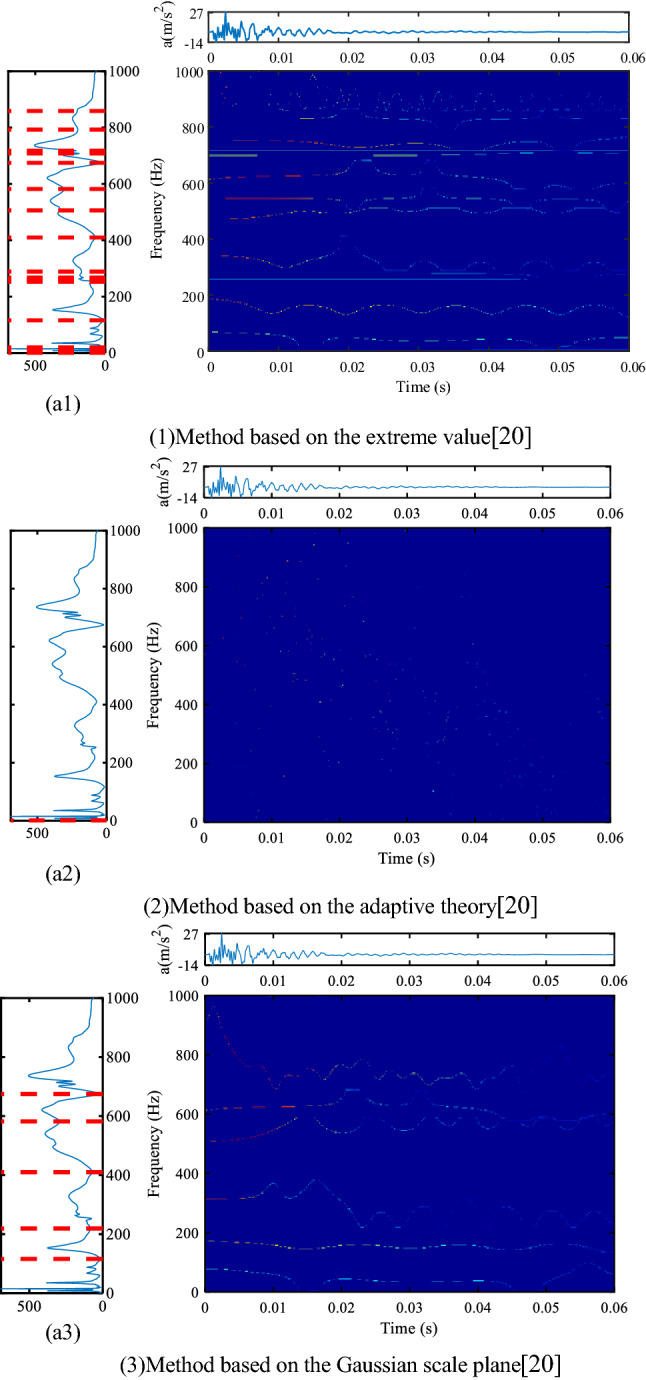
Spectrum segmentation and time–frequency display of hammering (K1) test data based on the traditional method, (**a1**–**a3**) is the Fourier spectrum segmentation results (upper left position, colormap, 'jet').

**Figure 6:**
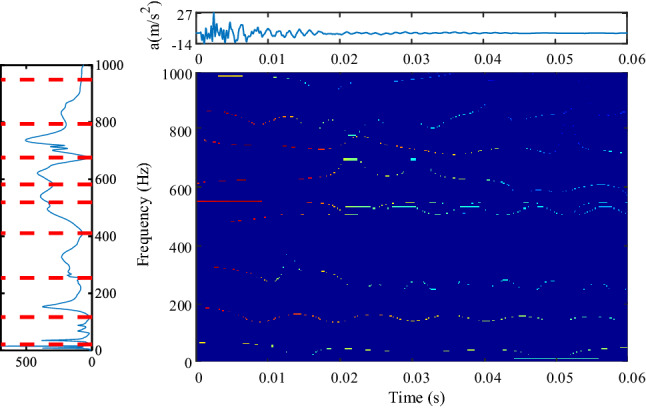
Spectrum segmentation and time–frequency display of hammering (K1) test data based on the method proposed in this paper, (**a**) is the Fourier spectrum segmentation results (upper left position, colormap, 'jet').

In the knock test, there is only the effect of instantaneous excitation, and no other vibration interference, so it is easy to observe the resonant frequency. The vibration waveform of transformer hammering test under normal state is shown in Figs. 5 and 6. For the resonance frequency component, we need to pay attention to the harmonic components of integral multiple of 50 Hz. These components are more likely to cause resonance in the transformer. The 'jet' colormap is used in Fig. 5 and all subsequent time–frequency figures.

Figure 5 shows the results of three traditional EWT methods based on extremum, adaptive and Gaussian plane^20^. In Fig. 5, the Fourier transform results and frequency domain segmentation are displayed on the left, the longitudinal representation is to facilitate comparison with the frequency components of the time–frequency plane, and the change of each frequency component with time is displayed on the right. The following Figs. 6, 8, and 9 are also the same.

The method based on extreme value needs to specify the number of extreme values in advance. When the number of specified extreme values is small, the division may be insufficient, which is similar to the division results based on adaptive method; when the number of extreme values is large, some frequency bands may be too narrow, too much bands will increase the burden of calculation, and too narrow bands will cause display distortion on the time–frequency plane, and some bright lines with unchanged color will appear, such as 250 Hz and 700 Hz in subfigure (1) of Fig. 5. The constant color means that the amplitude of these frequency components are constant, but in fact, these components are not always constant, because this is a hammer test, the vibration will decay quickly, so any frequency component cannot be unchanged. There is only one boundary in the range of 0–1000 Hz based on the adaptive method, the components to be analyzed are divided together, which is not helpful for transformer vibration analysis. The method based on the Gaussian plane is the best of the three methods. In addition to the resonance frequency components that can be seen in the method based on the Gaussian scale plane^20^, the method proposed in this paper can provide richer resonance frequencies, such as about 20 Hz, 550 Hz, 750 Hz and 850 Hz in Fig. 6. The proposed segmentation method can also give the resonant frequencies more accurately, that is, the line of the time–frequency plane is more straight. The method based on the Gaussian plane needs to draw a Gaussian plane when dividing the frequency domain, so the calculational burden is very heavy, the time–frequency analysis of the method based on Gaussian plane takes about 80 s, while the proposed method takes about 2 s. When time–frequency analysis is carried out for multiple channels, multiple hammering or faults, the method based on Gaussian plane needs high computational resources. Most importantly, the above three traditional methods cannot track and analyze the emerging frequency components.

In any case, EWT method is still a good time–frequency analysis method, especially the EWT method based on Gaussian plane. The method proposed in this paper is an improved EWT method. Compared with the method based on the Gaussian plane, the improved frequency domain segmentation method is simple in calculation and clear in the time–frequency display of important frequency components, which is more suitable for the analysis of transformers vibration data.

**Figure 7:**
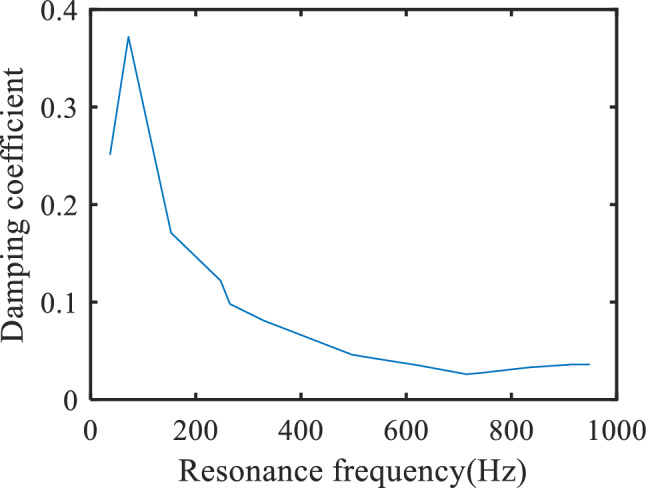
Variation of damping coefficient with resonance frequency.

Table 3 shows the identification results of resonance frequencies and damping coefficients corresponding to multiple resonance frequency points in Fig. 6. The change of transformer damping coefficient with resonance frequency is shown in Fig. 7. With the increase of resonance frequency, the damping coefficient shows a downward trend. For resonance frequency points above 400 Hz, the damping coefficients will be very small.

**Table 3:** Resonance frequency and correlation coefficient.

*ω*_*n*_ (Hz)	*X*	*ξ*
37	0.469	0.251
72	0.522	0.372
153	1.399	0.171
247	0.368	0.122
265	0.532	0.098
329	0.688	0.081
497	0.818	0.046
542	0.981	0.042
624	1.003	0.035
714	0.563	0.026
737	1.24	0.027
834	0.667	0.033
916	0.288	0.036
949	0.269	0.036

### Vibration analysis of transformer power on

When the hammering test of the transformer is inconvenient or impossible, the method of determining the resonant frequency of the transformer through the vibration data at power on is very useful. Especially for large power transformers, hammering test may be difficult to achieve. Since there is no current in the transformer winding when the transformer is powered on, it is unnecessary to consider the vibration caused by the load current, so the interference signal is small during the vibration measurement. When the transformer is powered on, it is equivalent to giving a physical excitation to itself. Although there are some vibration components generated by transformer excitation during no-load operation of transformer, however, the frequency spectrums of the excitation current are determined, so the vibration components generated by the excitation current are also determined. Therefore, the change of the resonance frequency can be observed by comparing the vibration spectrum when the transformer is powered on.

**Figure 8:**
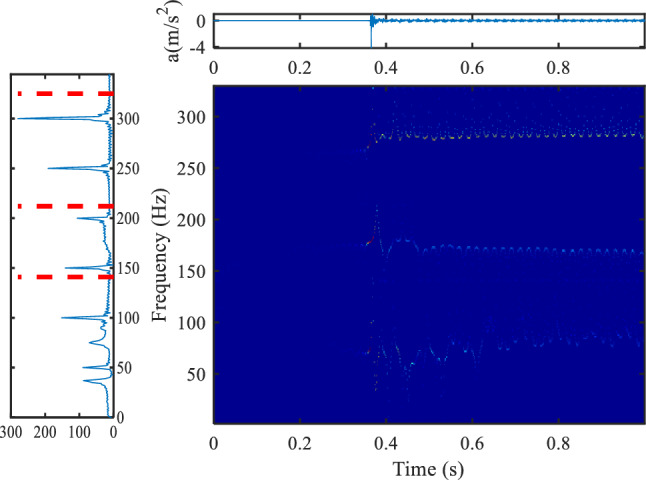
Vibration data analysis of transformer power on based on Gaussian plane^6^, (**a**) is the Fourier spectrum segmentation results (upper left position, colormap, 'jet').

**Figure 9:**
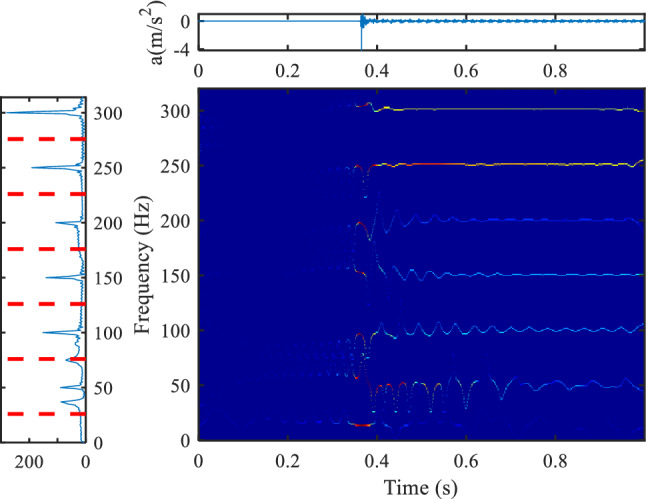
Vibration data analysis of transformer power on based on the method proposed in this paper, (**a**) is the Fourier spectrum segmentation results (upper left position, colormap, 'jet').

Figures 8 and 9 show the analysis results of the vibration data when the transformer is powered on, and the power on time of the transformer is 0.36 s. The method based on the Gaussian plane does not highlight the part with a large frequency amplitude, such as 100 Hz, 250 Hz, etc. The improved frequency domain segmentation results are shown in Fig. 9. It can be seen that the new segmentation method sets the boundary for the parts with large amplitude (100 Hz, 250 Hz). Compared with the results of the time–frequency plane in Fig. 8, the time–frequency information of the method proposed in this paper is more obvious.

**Figure 10:**
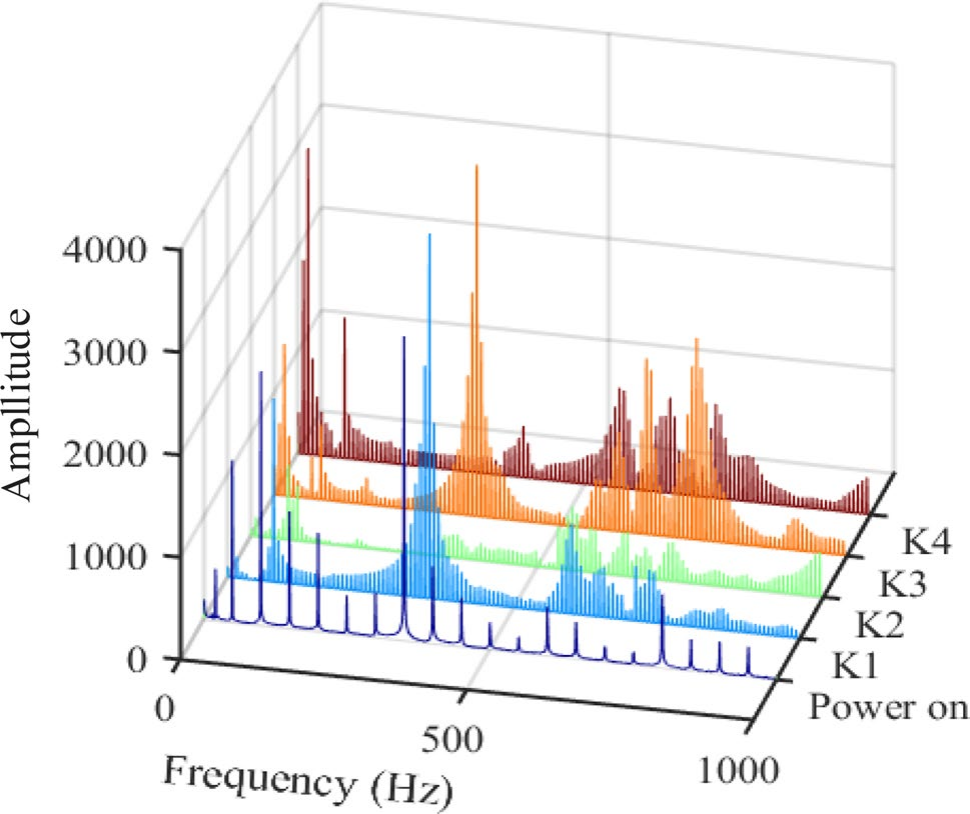
Vibration data analysis results of four hammering tests and power on (upper left position).

**Figure 11:**
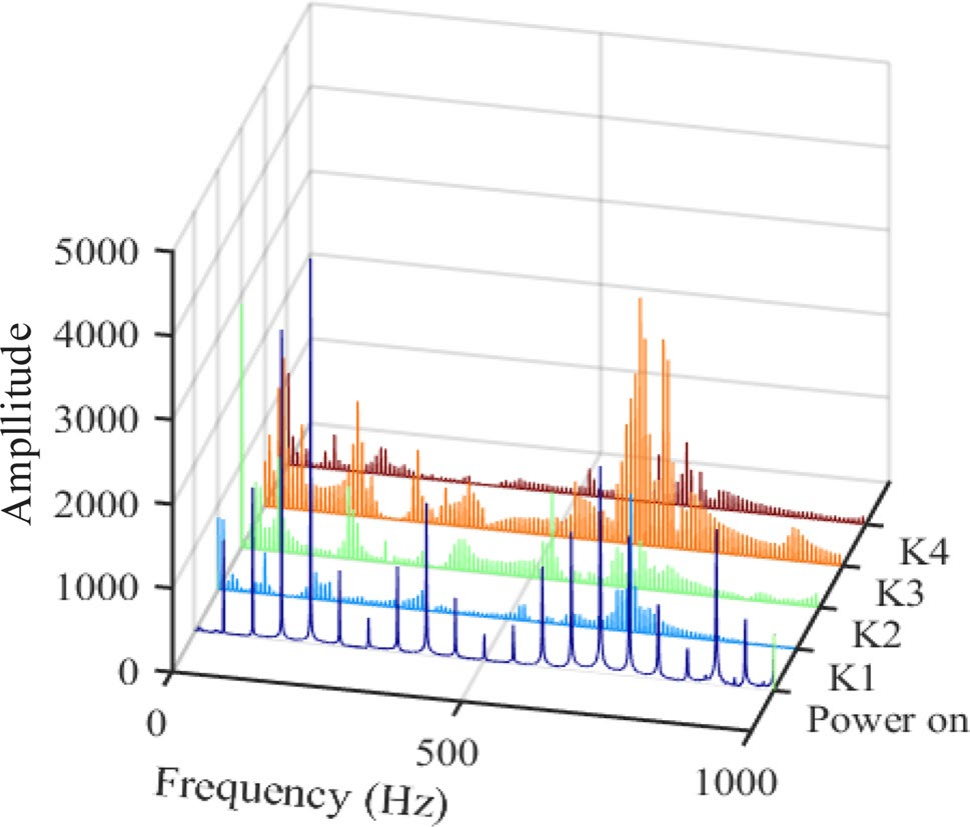
Vibration data analysis results of four hammering tests and power on (upper right position).

In Figs. 10 and 11, K1–K4 are Fourier analysis results of vibration of transformer hammering test, it can be seen that although the resonance frequencies excited by the four hammering tests are not exactly the same, the distribution range of resonance points is basically the same, and the resonance frequencies of the transformer can be obtained more completely by hammering in different directions. The first dark blue line is the analysis result when the transformer is powered on. It can be seen that the vibration components of the transformer include all frequency doubling components of 50 Hz within 0–1000 Hz in both transverse and longitudinal directions. Therefore, when a resonance point occurs within 1000 Hz, it will cause resonance on the frequency doubling component of 50 Hz adjacent to it. Except for the 100 Hz frequency point caused by the hysteresis, the resonant frequency points excited by the hammer test are basically the same as the vibration frequency points at power on. It can be seen that the transformer resonates at some frequencies, such as 350 Hz, 600 Hz and 800 Hz in Fig. 10 and 200 Hz, 400 Hz, 600 Hz ~ 800 Hz and 900 Hz in Fig. 11.

### Vibration analysis of transformer load step

**Figure 12:**
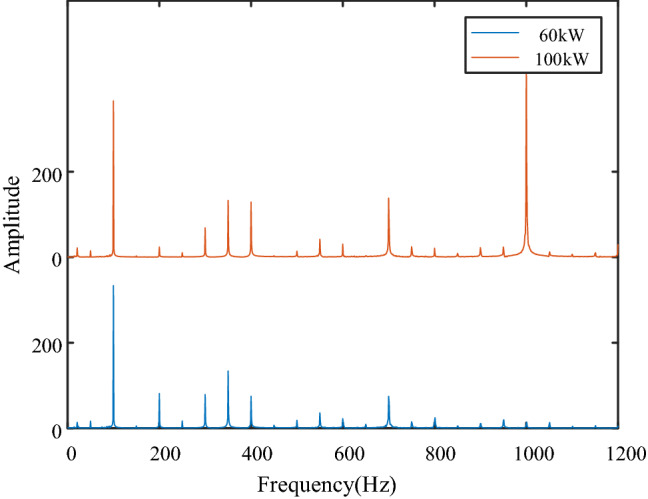
Frequency domain comparison before and after transformer load step (upper left position).

**Figure 13:**
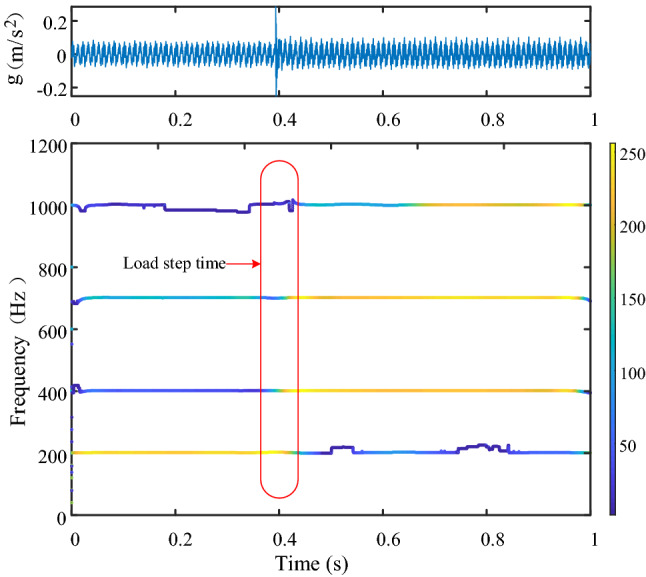
Vibration data and time–frequency display before and after transformer load step (upper left position).

Figure 12 shows the frequency spectrum of steady-state vibration of transformer under 60 kW and 100 kW load respectively. Figure 13 shows the vibration changes before and after the transformer load step, the load is switched from 60 to 100 kW, the load step time is about 0.39 s. By comparing the frequency spectrum of steady-state vibration before and after transformer load step, it can be seen that the components of 200 Hz, 400 Hz, 700 Hz and 1000 Hz change greatly, in which 1000 Hz component is an emerging frequency component. By setting the frequency domain division boundary near these components, it is obvious that the four frequency components change immediately after the load step, so the load step is the reason for the change of these frequency components.

When the transformer vibration data changes greatly, the components with large amplitude change are determined by comparing each signal component in the frequency domain, and then a boundary is set near the target frequency component to improve the time–frequency resolution of the target frequency and determine the occurrence time of the target frequency. Combined with the voltage and current signals of transformer load step, it can be determined whether the change of target frequency signal is caused by transformer faults or load step.

### Analysis of other transformers

**Figure 14:**
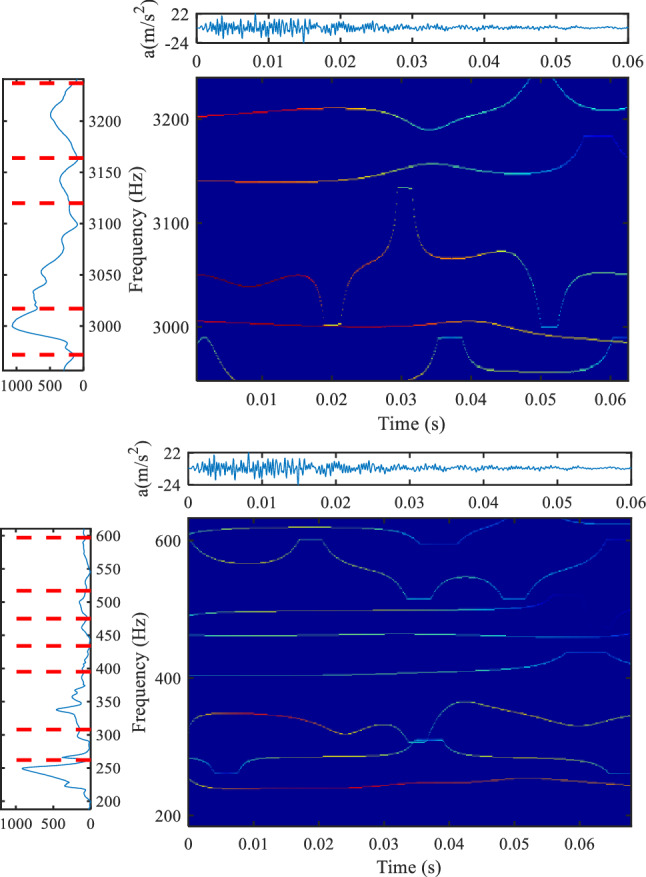
Analysis results of hammering test data of 25 kV oil immersed on-board transformer, (**a**) is the Fourier spectrum segmentation results (method proposed in this paper, upper left position, colormap, 'jet').

**Figure 15:**
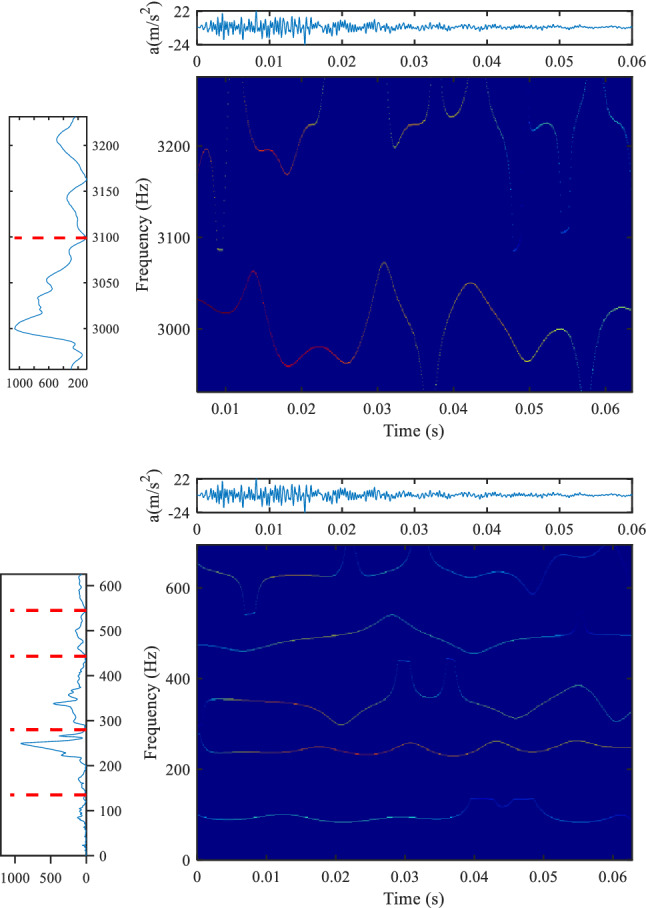
Analysis results of hammering test data of 25 kV oil immersed on-board transformer, (**a**) is the Fourier spectrum segmentation results (method based on the Gaussian scale plane, upper left position, colormap, 'jet').

Figures 14 and 15 show the analysis results of hammering test of 25 kV oil immersed on-board transformer for Electric Multiple Units (EMU). Figure 14 is the result based on the method proposed herein, and Fig. 15 is the result based on the conventional Gaussian plane. The spectrum distribution of 25 kV oil immersed on-board transformer is very wide, and there are many resonance frequency points in the range of 0–3000 Hz. The time–frequency analysis results of the high-frequency part and the low-frequency part are shown in Fig. 14. It can be seen that for the low-frequency part, both methods have better time–frequency resolution, and the proposed method is more densely divided; for the high-frequency part, the method proposed in this paper has obvious advantages, especially for the 3000 Hz component, the amplitude of the frequency component is very high, and the method based on the Gaussian plane does not show the change of the frequency component well.

**Figure 16:**
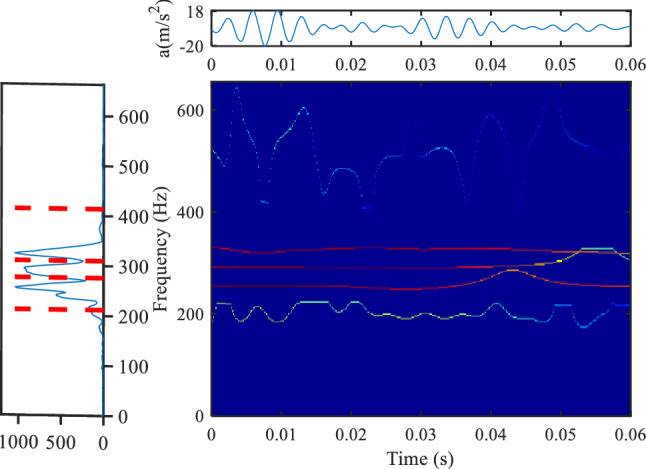
Analysis results of hammering test data of 35 kV metro traction transformer, (**a**) is the Fourier spectrum segmentation results (method proposed in this paper, upper left position, colormap, 'jet').

**Figure 17:**
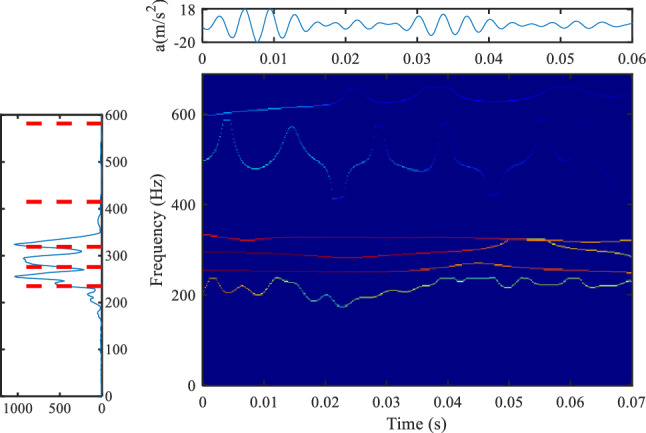
Analysis results of hammering test data of 35 kV subway dry-type transformer, (**a**) is the Fourier spectrum segmentation results (method based on the Gaussian scale plane, upper left position, colormap, 'jet').

Figures 16 and 17 are the analysis results of hammering test of 35 kV metro traction transformer. Figure 16 is the result based on the method proposed herein, and Fig. 17 is the result based on the conventional Gaussian plane. It can be seen that the resonance frequency distribution of dry-type transformer for 35 kV Metro is very concentrated, mainly in the range of 200–400 Hz, and both methods have good time–frequency resolution.

It can be seen that the method proposed in this paper has high time–frequency resolution for these two types of transformers. The method proposed in this paper has good applicability for both low-frequency components and high-frequency components, and the processing time for the vibration signals of the two types of transformers is less than 2 s. However, the method based on Gaussian plane has a running time of about 80 s.

Combined with the analysis results of three transformers, the advantages of the proposed method are as follows.

1. This method can track and analyze the sudden change component. When the vibration of transformer fluctuates, the cause of vibration fluctuation can be determined by locating the frequency change time and load step time, which is of great significance to realize the on-line fault diagnosis of transformer.
2. Through the proposed method, the vibration waveforms at resonance frequencies are separated, and the damping coefficients and other correlation coefficients at different resonance frequencies are extracted.
3. For large power transformers, hammering test may be difficult to achieve. When the hammering test of the transformer is inconvenient or impossible, the resonance frequencies of the transformer can be determined by the vibration data during no-load power on.

The disadvantage of this method is that for different types of transformers, the determination of *k*_*p*_ (in Eq. 24) and *k*_*q*_ (in Eq. 25) needs to be combined with the vibration of the transformer and adjusted accordingly according to the analysis objectives.

## Conclusion

An improved EWT method is proposed in this paper. Through the analysis of vibration signals of 380 V dry-type transformer, 35 kV subway dry-type transformer and 25 kV oil immersed on-board transformer of EMU, the applicability of this method for transformers with different voltage levels and different capacities is proved.

The method proposed in this paper can effectively eliminate the influence of interference components such as small amplitude components and multiple adjacent high amplitude components, and highlights the importance of high amplitude components and components with large changes in frequency domain. Most importantly, compared with traditional methods, the proposed method can track and analyze the new frequency components. Through the analysis of transformer hammering vibration data and power on vibration data, this method has higher time–frequency resolution, and the calculation time is shortened from 80 s to about 2 s, which proves the superiority of this method.

## Supplementary Information

Supplementary Information.

## Data Availability

All data generated during this study are included in this published article [and its supplementary information files].

## References

[1] Dai J, Song H, Sheng G, Jiang X (2017). Dissolved gas analysis of insulating oil for power transformer fault diagnosis with deep belief network. IEEE T. Dielect. El. In..

[2] Zhang B, Yan N, Du J, Han F, Wang H (2018). A novel approach to investigate the core vibration in power transformers. IEEE T. Magn..

[3] Bagheri M, Zollanvari A, Nezhivenko S (2018). Transformer fault condition prognosis using vibration signals over cloud environment. IEEE Access.

[4] Lee SH, Jung SY, Lee BW (2010). Partial discharge measurements of cryogenic dielectric materials in an HTS transformer using HFCT. IEEE T. Appl. Supercon..

[5] Yang Z, Zhou Q, Wu X, Zhao Z (2019). A novel measuring method of interfacial tension of transformer oil combined PSO optimized SVM and multi frequency ultrasonic technology. IEEE Access.

[6] Kim JW, Park B, Jeong SC, Kim SW, Park P (2005). Fault diagnosis of a power transformer using an improved frequency-response analysis. IEEE T. Power Deliver..

[7] Zhang P, Li L, Cheng Z, Tian C, Han Y (2019). Study on vibration of iron core of transformer and reactor based on maxwell stress and anisotropic magnetostriction. IEEE T. Magn..

[8] Saponara S, Fanucci L, Bernardo F, Falciani A (2016). Predictive diagnosis of high-power transformer faults by networking vibration measuring nodes with integrated signal processing. IEEE T. Instrum. Meas..

[9] Kang P, Birtwhistle D (2001). Condition monitoring of power transformer on-load tap-changers. Part 2: Detection of ageing from vibration signatures. IEE Proc. Generation Transmission Distribution..

[10] Garcia B, Burgos JC, Alonso A (2006). Transformer tank vibration modeling as a method of detecting winding deformations—Part I: Theoretical foundation. IEEE T. Power Deliver..

[11] Shin PS, Lee J, Ha JW (2007). A free vibration analysis of helical windings of power transformer by pseudospectral method. IEEE T. Magn..

[12] Shengchang J, Yongfen L, Yanming L (2006). Research on extraction technique of transformer core fundamental frequency vibration based on OLCM. IEEE T. Power Deliver..

[13] Hsu C, Lee S, Lin C, Liu C, Chang S, Hsieh M, Huang Y, Fu C (2015). Reduction of vibration and sound-level for a single-phase power transformer with large capacity. IEEE T. Magn..

[14] Jing Z, Hai H, Pan J, Yanni Z (2016). Identification of the nonlinear vibration system of power transformers. Meas. Sci. Technol..

[15] Bartoletti C, Desiderio M, DiCarlo D, Fazio G, Muzi F, Sacerdoti G, Salvatori F (2004). Vibro-acoustic techniques to diagnose power transformers. IEEE T. Power Deliver..

[16] X. Zhang, R. Huang, D. Zhou, F. Wang (2018) Vibration monitoring of converter transformer by simplified permutation entropy, 2018. IEEE, 864–869. 10.1109/CAC.2018.8623221

[17] Luo Bo, Wang F, Liao T, He Z (2014). Modal parameters identification of power transformer winding based on the improved complex Morlet wavelet. J. Vib. Shock.

[18] Yan O, Lin X, Yin J (2007). Features of vibration data of power transformer using the wavelet theory. High Voltage Eng..

[19] Duan R, Wang F (2016). Fault diagnosis of on-load tap-changer in converter transformer based on time-frequency vibration analysis. IEEE T. Ind. Electron..

[20] Gilles J (2013). Empirical wavelet transform. IEEE T. Signal Proces..

[21] Liu W, Cao S, Chen Y (2016). Seismic time-frequency analysis via empirical wavelet transform. IEEE Geosci. Remote S..

[22] Liu N, Li Z, Sun F, Wang Q, Gao J (2019). The improved empirical wavelet transform and applications to seismic reflection data. IEEE Geosci. Remote S..

[23] Parashar D, Agrawal DK (2021). Automatic classification of glaucoma stages using two-dimensional tensor empirical wavelet transform. IEEE Signal Proc. Let..

[24] Xin Y, Li S, Zhang Z, An Z, Wang J (2019). Adaptive reinforced empirical morlet wavelet transform and its application in fault diagnosis of rotating machinery. IEEE Access.

[25] Lu S, Gao W, Hong C, Sun Y (2021). A newly-designed fault diagnostic method for transformers via improved empirical wavelet transform and kernel extreme learning machine. Adv. Eng. Inform..

[26] Zhao M, Xu G (2018). Feature extraction of power transformer vibration signals based on empirical wavelet transform and multiscale entropy. IET Sci. Meas. Technol..

