# Supplementary material for: Improved empirical wavelet transform (EWT) and its application in non-stationary vibration signal of transformer
Source: Sci Rep. 2022 Oct 20;12:17533. doi: 10.1038/s41598-022-22519-z (PMC9584928; doi:10.1038/s41598-022-22519-z)
Supplement: Supplementary file 1 — Supplementary Information. [file 41598_2022_22519_MOESM1_ESM.zip › Data Description.docx]

1. For the 380V transformer, that is, the transformer in Fig. 4 in the paper, there are two data files, the test file of knocking vibration (KnockVibrationDryTransformer380V) and the vibration test file during operation (RunVibrationDryTransformer380V). In these two files, there are vibration signals of two channels, the vibration of the upper left position and the vibration of the upper right position. The vibration of the upper left position is named Data1_ AI_ 1__ 1_____ AI_ 1. The upper right channel is named Data1_ AI_ 2__ 2_____ AI_ 2. The sampling rate is 16384Hz.
2. For 25kV and 35kV transformers, there is only one vibration test signal, and the sampling rates are 16384Hz and 6250Hz respectively
